# Acute appendicitis: position paper, WSES, 2013

**DOI:** 10.1186/1749-7922-9-26

**Published:** 2014-04-07

**Authors:** Ferdinando Agresta, Luca Ansaloni, Fausto Catena, Luca Andrea Verza, Daniela Prando

**Affiliations:** 1 Department of General Surgery, ULSS19 del Veneto, Piazzale Etruschi, 9, Adria 45011, RO, Italy; 2 General Surgery I, Papa Giovanni XXIII Hospital, Bergamo, Italy; 3 Department of Emergency Surgery, University of Parma, Parma, Italy

## Abstract

Appendectomy is one of the most frequently performed operative procedures in general surgery departments of every size and category. Laparoscopic Appendectomy – LA - as compared to Open Appendectomy – OA - was very controversial at first but has found increasing acceptance all over the World, although the percentage of its acceptance is different in the various single National setting. Various meta-analyses and Cochrane reviews have compared LA with OA and different technical details. Furthermore, new surgical methods have recently emerged, namely, the single-port/incision laparoscopic appendectomy and NOTES technique. Their distribution among the hospitals, however, is unclear. Using laparoscopic mini-instruments with trocars of 2–3.5 mm diameter is proposed as a reliable alternative due to less postoperative pain and improved aesthetics. How to proceed in case of an inconspicuous appendix during a procedure planned as an appendectomy remains controversial despite existing study results. But the main question still is: operate or not operate an acute appendicitis, in the meaning of an attempt of a conservative antibiotic therapy. Therefore, we have done a literature survey on the performance of appendectomies and their technical details as well as the management of the intraoperative finding of an inconspicuous appendix in order to write down – under the light of the latest evidence – a position paper.

## Serch strategy

Literature research for the Consensus update on laparoscopic appendectomy followed the following criteria: Guidelines (1990–2013) on the argument were taken in consideration, including references cited in the papers or web pages; PubMed has been searched, at first, with the following criteria: ***Limits Activated*:** Humans, Clinical Trial, Meta-Analysis, Practice Guideline, Randomized Controlled Trial, Review, English, All Adult: 19+ years, published in the last 5 years; *Search details*: [(("laparoscopy" [MeSH Terms] OR "laparoscopic" [All Fields]) AND ("appendectomy" [MeSH Terms] OR "appendectomy" [All Fields])) AND ("humans" [MeSH Terms] AND (Clinical Trial [ptyp] OR Meta-Analysis [ptyp] OR Practice Guideline [ptyp] OR Randomized Controlled Trial[ptyp] OR Review [ptyp]) AND English [lang] AND "adult" [MeSH Terms] AND "2005/1/1" [PDat]: "2013/04/30" [PDat])]. Cross-link control was performed with EMBASE, Google Scholar and Cochrane library databases. The Oxford 2011 Levels of Evidence ( http://www.cebm.net/index.aspx?o=5653) has been used to rank the level of evidence (LE) to the article cited.

After Semm performed the first LA in 1980 [1], this new technique was picked up at the beginning only slowly, with an increase in its use mainly after the 2005. Meanwhile, there are a number of meta-analyses, prospective randomized trials, and Cochrane analyses comparing LA, OA, and different details concerning the operative procedure itself. However it remains unclear how far and if the recommendations reported are being adapted in clinical practice [2-5]. In a Sauerland’s Cochrane analysis [6] (LE 1), the rate of wound infections, the first postoperative day’ pain, hospital stay, postoperative return to solid food, first postoperative bowel movement, surgery-related aesthetics, and return to normal activity were significantly better after LA as compared to OA. On the other side, the rates of intraabdominal abscesses, procedural time, and the costs of LA and its overall hospital-related costs were significantly higher, although the costs after discharge from the hospital were significantly lower for LA. The costs related to the surgical procedure itself greatly depend on the surgeon’s choice for type of trocar and the technique for control of the mesoappendix and the appendix stump. In a paper by Chu [7], these three factors alone affect costs to vary between $81 and $873. Despite the partly marginal advantages and a limited clinical relevance, Sauerland et al. recommended the laparoscopic technique. Especially young, female, obese, and working patients seem to profit from this technique. A further Cochrane review by Guitan [8] (LE 1) has confirmed the recommendation of LA especially for fertile women due to a higher diagnostic value when compared to OA and a lower rate of resection of inconspicuous appendices, although the rate of adverse events has not been reduced. All the advantages of LA versus OA has also been confirmed also by a recent meta-analysis of 25 studies including 2,220 LAs and 2,474 OA, especially concerned less postoperative complications and pain, an earlier return to food intake, a shorter hospital stay, and an earlier return to work and normal activity. Another interesting point reported in this analysis is that hospital-related costs were not differ significantly between the two procedures, although the LA surgical time was significantly longer [9] (LE I).

The European Association for Endoscopic Surgery recommends LA in their evidence-based guidelines for the treatment of suspected acute appendicitis due to a significantly lower rate of wound infections and quicker postoperative recovery [10]. The Society of American Gastrointestinal and Endoscopic Surgeons, too, recommends LA in different patient collectives [11]. Two further Italians guidelines [12,13] on the same topic recommend the laparoscopic approach in both uncomplicated as complicated appendicitis, but above all in both these guidelines has been stressed the idea of laparoscopy as a final diagnostic and formal therapeutic act (LE I). It is also well pointed out the idea that, has previously reported in the EAES guidelines [10], the converted cases have similar outcome when compared to primarily open cases (LE II). Besides fertile women, groups at major risk of complications, such as elderly and obese patients, would benefit most from a laparoscopic approach [14-24] (LE III). It is interesting to notice that about this two groups of patients - elderly and obese – have beer recently published two papers were the National Surgical Quality Improvement Program database has been used. In the one by Mason et al. [25], 13330 obese patients (body mass index ≥ 30) who underwent an appendectomy (78% LA, 22% OA) during the period 2005–2009, have been identified and their short-term outcomes has been analysed, using the American College of Surgeons National Surgical Quality Improvement Program database. The Conclusions of the Authors is that the analysis of the NSQIP database showed that the LA is superior to the OA in obese patients and that a considerably greater risk of complications is associated with the open technique; most of the morbidity is due to wound-related issues that become more prevalent in the open approach with increasing obesity. In addition, length of stay (LOS) and operative times were considerably lower in patients approached laparoscopically, potentially reducing hospital costs. Nevertheless, despite the added benefits of laparoscopy in patients with complicated appendicitis, use of the laparoscope was low in this group of obese patients. Moazzez et all [26], still using the American College of Surgeons National Surgical Quality Improvement Program (ACS/NSQIP) databases for years 2005–2009, has identified 3,674 patients (age over 65 years) who underwent an appendectomy for appendicitis, of whom 72% with LA. The Authors conclusions is that, through aggregate and matched cohort analysis of elderly patients who underwent an OA or LA for appendicitis, this last one was associated with less minor and overall morbidity and lower superficial Surgical Site Infection and a shorter LOS.

Regarding appendiceal stump closure, a meta-analysis compared staplers versus the endoloop technique for LA [27]. A significant advantage for stapler appendectomy was found for wound infections and postoperative ileus (LE I), but this meta-analysis has not confirmed the significantly lowered rate of intraabdominal abscesses and readmissions that were reported elsewhere in the literature [28] (LE IV) One bias to take in consideration when reading a large case series published on the subject is that the use of stapler devices was mainly used for extensive inflammation, i.e., in cases with a higher risk of infection [28] (LE IV).

Two novel ways of the abdominal access route, the single-port/incision laparoscopic appendectomy (SPILA) technique and NOTES (natural orifice transluminal surgery), have emerged in recent years. The German Society for General and Visceral Surgery (DGAV) started the national NOTES registry for NOTES procedures (including appendectomies) in February 2008 [29]. The SPILA is supposed to avoid visible scars by introducing all instruments through a single port at the umbilicus. Although the results reported in the Literature seem to be positive (the incidence of complications with SPILA remains low and operating times between new and traditional approaches are comparable), articles retrieved varied in quality, generally representing low-level evidence, at high risk of intrinsic bias. The literature fails also to formally document cosmetic results using questionnaires or visual assessment scales, thus preventing assessment of this outcomes. Adequately randomized trials are required to assess the real effectiveness of the SPILA [30] (LE I).

The same difficulties occur with the NA: This approach nowadays is admitted only in strictly controlled and experimental protocols [12].

Needlescopy might be applied only in selected and not complicated cases due to its higher rate of conversions and prolonged OT time [31] (LE I).

Another very important point is the management of the intraoperative finding of an inconspicuous appendix during an operation for suspected appendicitis. In the absence of an intraabdominal pathological finding explaining the symptoms, the appendectomy "en principe" is recommended due to the high rate of histologically found appendicitis - so called endoappendicites - despite a macroscopically normal appearance in up to 26% of cases [32] (LE V). Considering also that morbidity of appendectomy does not significantly exceed that of the explorative laparoscopy [12].

Operate or not operate an acute appendicitis? That’s the (main) question, someone could say. Although there are some evidence in literature of the role of an attempt with a conservative antibiotic therapy in case of a suspicious of an acute appendicitis (when perforation and peritonitis is not suspected) in selected patients, the problem is how to select them. Although Antibiotic therapy is associated with up to 70% success rate and a trend toward decreased risk of complications without prolonging hospital stay, however, no conclusion is possible to write down according to the available literature due to its low methodological quality [33] (LE II). While waiting for the results of some prospective trial on this topic, actually there are no doubts to agree with what Ansaloni and coll. have written in their paper "*…Conservative antibiotic therapy for AA should continue to be considered within the limitations imposed by its inherent advantages and disadvantages; surgery remains the gold standard for treating AA despite the clinical challenges involved…".*[34] (LE III).

In a frame time of economic problems all around the world, it is a must to take a position according the cost of LA. It is hard to state anything that could apply everywhere, first because obviously the direct cost (operating room occupancy longer?; instruments etc.) of a LA is more than that of an OA and second because LA can be performed using a myriad of techniques, the cost of each method varies (range from US $81 to US $873). Concerning the first point (LA versus OA), although it could sound philosophy, the indirect cost of the LA (less pain, less morbidity, less length of hospital stay, faster return to daily activity and so on) are surely less of the OA ones. About the second we do agree with Chu and coll: *"… surgeons should review the cost implications of their practice and to find ways to provide the most costeffective care without jeopardizing clinical outcome…"*[7].

## Competing interests

The authors declare that they have no competing interests.

## Authors’ contributions

FA drafted the manuscript. FA, LA, FC, LAV, DP reviewed the draft and made corrections and revisions. All authors read and approved the final manuscript.
